# Erythrocyte sequestration of metformin in horses: impact on matrix-specific pharmacokinetics and detection windows

**DOI:** 10.1186/s12917-026-05449-0

**Published:** 2026-04-16

**Authors:** Megan E. Jacobs, Jeff Blea, Michael Hardy, Daniel S. McKemie, Megan Traynham, Heather K. Knych

**Affiliations:** 1 K.L. Maddy Equine Analytical Chemistry Laboratory (Pharmacology Section), University of California, Davis, School of Veterinary Medicine, 620 West Health Science Drive, Davis, CA 95616 USA; 2 School of Veterinary Medicine, University of California, Davis, USA; 3 Racing Medication and Testing Consortium, Lexington, KY USA; 4 Department of Molecular Biosciences, School of Veterinary Medicine, University of California, Davis, USA

**Keywords:** Metformin, Erythrocyte partitioning, Matrix effect, Detection window, Pharmacokinetics, Performance horse

## Abstract

**Background:**

Metformin is used for the treatment of type 2 diabetes and is one of the most prescribed medications in human medicine. It is less commonly prescribed in equine medicine, and its use is tightly regulated in several performance horse disciplines. In horseracing, it is considered a banned substance. A previous pharmacokinetic study of metformin in horses demonstrated a prolonged, unpredictable elimination phase. In the current study it was hypothesized that this was due to sequestration of metformin in a “deep” compartment, specifically red blood cells. This could result in pharmacokinetic differences between blood matrices (i.e. blood and serum). The objective of the current study was to assess red blood cell partitioning and the concentrations of metformin in different blood matrices following oral administration to horses. To that end, six horses received a single 15 g oral dose of metformin and plasma, serum, whole blood, red blood cells, and urine samples were collected starting at 5 min until 31 days post administration. Concentrations of metformin were determined using liquid chromatography-tandem mass spectrometry, and pharmacokinetic analysis performed.

**Results:**

Red blood cells act as a reservoir for metformin in horses with the average blood to plasma ratio ranging from < 1 to > 10 at the later time points. The terminal half-life (mean ± SD) was 14.7 ± 7.25, 75.4 ± 32.2 and 49.1 ± 7.01 in plasma, serum, and red blood cells, respectively. The difference between serum and plasma concentrations was > 15% at several time points, especially at the later times. Concentrations in urine samples, fluctuated unpredictably over time.

**Conclusion:**

Red blood cells act as a reservoir for metformin leading to a prolonged detection time, necessitating an extended withdrawal time for oral administration prior to competition in performance horses to prevent inadvertent positive drug tests. Additionally, differences in metformin concentrations across various biological matrices may preclude the extrapolation of data from one sample type to another.

**Supplementary Information:**

The online version contains supplementary material available at 10.1186/s12917-026-05449-0.

## Introduction

Metformin (1,1-dimethylbiguanide) is a medication commonly used in the treatment of type 2 diabetes mellitus in humans. It works by lowering hepatic glucose production and increasing peripheral tissue glucose sensitivity to lower overall blood glucose concentrations [1]. It is a small, hydrophilic molecule and primarily excreted in the urine as metformin [2]. As a result of its effectiveness and wide safety margin, metformin is one of the most commonly prescribed drugs in human, medicine [3].

In contrast to its widespread use in human medicine, metformin is less commonly prescribed in veterinary medicine. While it is sometimes used for treating insulin dysregulation in horses, its efficacy is controversial. One study found that metformin did not improve insulin sensitivity in insulin-resistant ponies following oral administration [4]. This is likely attributable, at least in part, to its low bioavailability in horses (~ 8%) [5, 6]. In horses, similar to humans, metformin is excreted in the urine as the parent compound [6].

Although metformin is not commonly administered to horses, recent reports of positive drug tests in performance horses, necessitates a thorough understanding of the behavior of this drug. The pharmacokinetics of metformin in horses have been reported [5, 6]. In a recent study, metformin pharmacokinetics were studied in 12 horses following a single oral administration of 15 g and an IV dose of 5 mg/kg [6]. Metformin concentrations remained above the limit of detection (LOD) of the analytical assay (0.1 ng/mL) for 21 days in blood and > 80 days in urine [6]. The terminal half-life ranged from 56.5–111.0 h [6]. In humans, there is evidence that metformin partitions into and sequesters in red blood cells (RBC) [7]. The drug is then slowly released back into the plasma, resulting in an extended residence time in the body [7]. If this same phenomenon occurs in horses, this may explain the prolonged detection time and long terminal half-life in this species. In addition, for drugs that exhibit significant sequestration in RBCs, concentrations can differ between blood matrices. For example, plasma and serum concentrations may not be comparable, primarily because of the way the samples are processed. Given that different performance horse disciplines regulate drugs in different matrices, this can have regulatory implications. Therefore, understanding how a drug behaves is imperative for appropriate regulation.

To that end, the primary objective of the current study was to determine if metformin partitions into RBCs by measuring metformin concentrations in different blood matrices and the effect of RBC hemolysis on serum and plasma concentrations. A secondary objective was to describe metformin concentrations and determine pharmacokinetic parameters in different blood matrices, to provide data to appropriately regulate administration of this drug to performance horses.

## Methods

### Animals

Six healthy, university-owned Thoroughbred horses (4 geldings, 2 mares; weighing 473–604 kg, aged 3–6 years) were used for this study. Health status was assessed by physical examination, complete blood count, and serum biochemistry panel prior to beginning the study. Horses did not receive any medications for 2 weeks prior to commencement of the study. Horses were housed in 12 × 24-foot pens and exercised 5 days a week using a Equineciser (Centaur Horse Walkers Inc, Mira Loma, CA, USA) and a high-speed treadmill (Mustang 2200, Graber AG, Switzerland) following standard protocols established by our laboratory [8]. Horses were exercised prior to and for the duration of the study, with the exception of the day of drug administration. Food and water were available ad libitum for the entirety of the study, except for the 12 h directly prior to and for 2 h post drug administration, when food was withheld. This study was approved by the Institutional Animal Care and Use Committee of the University of California, Davis (Protocol # 23,732).

### Instrumentation and drug administration

A 14-gauge catheter was placed aseptically in the external jugular vein of all horses for blood sample collection. Horses received a single oral administration of 15 g (25–32 mg/kg) of metformin. Metformin tablets (Aurobindo Pharma, Hyderabad, Telangana, India) were crushed and suspended in water in a dosing syringe. Drug was administered directly into the oral cavity within 1 h of formulation. Sampling catheters were removed 18 h after dosing, and all following samples were collected via direct venipuncture.

### Sample collection and processing

**Fig. 1:**
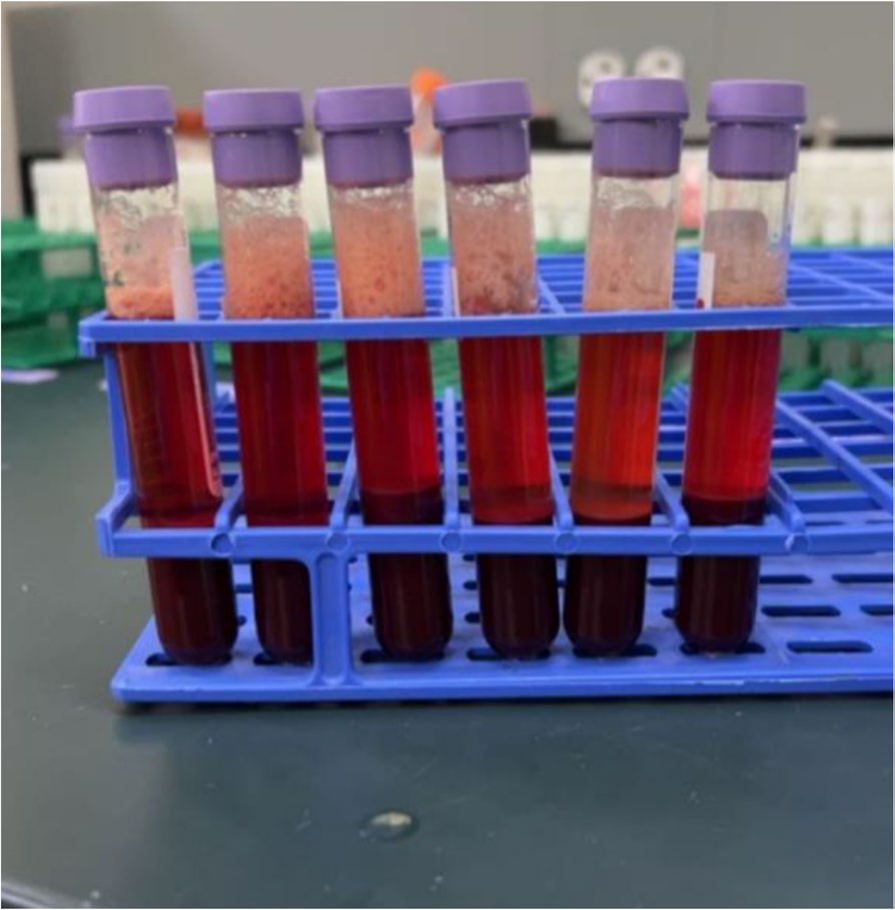
Example of hemolyzed plasma samples collected post metformin administration

Blood samples were collected into Ethylenediaminetetraacetic acid (EDTA) blood tubes for determination of metformin concentrations in plasma, RBCs, and whole blood. For determination of metformin concentrations in serum, one sample was collected into a blood tube devoid of anticoagulant and one sample was collected into a tube containing a serum separator (SST). Since metformin partitions into RBCs in other species, one additional plasma sample (EDTA) and one additional serum sample (without serum separator) were collected to assess whether hemolysis of red blood cells could lead to the release of metformin into the serum or plasma fraction of the blood samples. Samples were collected at times 0 (prior to drug administration), 5, 10, 15, 30, 45 min, and 1, 1.5, 2, 3, 4, 5, 6, 8, 12, 18, 24, 30, 36, 48, 72, 96, 120, 144, 168 h, and 9, 11, 14, 16, 20, 22, 24, 27, 29, and 31 days post drug administration. Immediately following sample collection, the blood samples from one EDTA tube and one tube devoid of anticoagulant were repeatedly drawn up and reinserted back into the tubes with a clean syringe to induce hemolysis (Fig. 1). Following collection, all samples in EDTA tubes were kept on ice. The tubes devoid of anticoagulant and the SST were kept at room temperature. All samples were processed within 1 h of collection.

An aliquot of whole blood was taken from one EDTA tube, while the other 2 EDTA tubes were centrifuged at 3000 g at 4 °C for 10 min. One aliquot of plasma (one hemolyzed, one non-hemolyzed) was taken from each tube, as well as an aliquot of RBCs from the non-hemolyzed tube. The 2 tubes devoid of anticoagulant and SST were incubated for 30 min to 1 h at 37 °C, then centrifuged at 3000 g for 10 min. An aliquot of serum was taken from each tube (1 hemolyzed, 2 non-hemolyzed). All samples were stored at −20 °C until drug concentration analysis.

As horses are trained to urinate on command, urine was collected via free catch. Samples were collected at 24, 48, 72, 96, 120, 144, 168 h, and 9, 11, 14, 16, 20, 22, 24, 27, 29, 31, 34, 36, and 41 days post drug administration. Samples were kept on ice and subsequently stored at −20 °C until drug concentration analysis.

### Drug concentration determination

For both blood and urine samples, calibration curves, quality control samples, and negative control samples were prepared fresh for each assay. Metformin stock solutions (Cerilliant, Round Rock, TX) were generated by diluting working standard solutions with methanol. Fifteen calibrators for each blood sample type were prepared by dilution of the working standard solutions with drug free matrix to concentrations ranging from 0.25 to 5,000 ng/mL. Similarly, urine calibrators (13 calibrators between 0.5 and 25,000 ng/mL) were prepared by dilution of the working standard solutions with drug free equine urine. Quality control samples for blood were prepared at concentrations of 0.75, 40, and 5000 ng/mL by fortifying drug free matrices with analyte. For urine, quality control samples were prepared by fortifying drug free urine with analyte at 0.75, 100, and 5000 ng/mL.

Serum samples were processed and extracted as described previously [6]. For RBC lysis in RBC and whole blood samples, 0.5 mL of sample was combined with 0.5 mL water, mixed for 1 min, incubated for 10 min at room temperature, and mixed for an additional 1 min prior to freezing at −80 ºC. Samples were thawed at room temperature and 0.5 mL was aliquoted to a new vial. Whole blood, RBCs, and plasma samples underwent protein precipitation as described previously for serum samples [6]. Urine samples were extracted as previously described [6].

For all matrices, 20 µL was injected into the liquid chromatography tandem mass spectrometry (LC–MS/MS) system and quantitative analysis was conducted as previously described [6].

The limit of quantitation (LOQ; the lowest calibrator that could be measured with acceptable precision) was 0.25 ng/mL for plasma and serum, and 0.3 ng/mL for RBCs and whole blood. The LOD (lowest calibrator with a 3:1 signal to noise ratio) was 0.1 ng/mL for plasma and serum and 0.2 ng/mL for RBCs and whole blood. For urine the LOQ and LOD were 0.5 ng/mL and 0.25 ng/mL, respectively.

Quality control samples met accuracy and precision acceptance criteria as described in the Food and Drug Administration’s (FDA) Guidance for Bioanalytical Method Development and Validation [9] (Table 1).

**Table 1:** Accuracy and precision values for LC–MS/MS analysis of metformin in equine serum, plasma, RBCs, whole blood, and urine

Matrix	Concentration (ng/mL)	Accuracy (% nominal concentration)	Precision (% relative SD)
Serum			
	0.75	91.0	12.0
	40.0	101	1.0
	5000	100	2.0
Plasma			
	0.75	106	4.0
	40.0	106	1.0
	5000	107	3.0
RBCs			
	0.75	98.0	13.0
	40.0	108	1.0
	5000	101	2.0
Whole Blood			
	0.75	100	9.0
	40.0	104	2.0
	5000	99.0	4.0
Urine			
	0.75	92.0	6.0
	100	105	6.0
	5000	95.0	3.0

#### In vitro assessment of matrix comparability (Plasma vs Serum)

An in vitro study was conducted to compare metformin concentrations in plasma and serum. Blood samples collected from six horses not administered metformin were compared in vitro. For each horse, three tubes were collected: (1) EDTA blood tube, (2) tube devoid of anti-coagulant, and (3) tube devoid of anticoagulant but with a serum separator. Three aliquots were taken from each tube type for each horse. Each aliquot was fortified with metformin at the low and high end of the calibration curve. Samples were processed and analyzed for determination of metformin concentrations as described for administration samples.

Comparability between plasma and serum was assessed by calculating the % relative difference with plasma concentration as the reference matrix, as plasma is the standard matrix for pharmacokinetic characterization of metformin. The percent relative difference was calculated for each paired sample at each time point using the formula:

$$\%Relative Difference= \frac{{C}_{serum}-{C}_{plasma}}{{C}_{plasma}} x 100$$

To calculate the % relative difference between serum samples collected in tubes without a separator compared to serum collected in SSTs, serum samples from tubes without a separator were used as the reference matrix with the formula:

$$\%Relative Difference= \frac{{C}_{serum}-{C}_{SST}}{{C}_{serum}} x 100$$

where C_serum_ is serum collected in tubes without a separator and C_SST_ is serum collected in SSTs.

A mean bias < ± 15% was considered an indication of comparable concentrations between the matrices in alignment with international regulatory guidelines for bioanalytical cross-validation and analytical accuracy [10].

### Concentration and pharmacokinetic analysis

Metformin concentrations at each timepoint post administration were compared between plasma and serum samples and between serum samples collected in SST tubes and tubes without a separator as described above for “Assessment of Matrix Comparability.” The blood to plasma (B/P) ratio was calculated using the formula:

$$B/P= \raisebox{1ex}{${[metformin]}_{whole blood}$}\!\left/ \!\raisebox{-1ex}{${[metformin]}_{plasma}$}\right.$$

Non-compartmental analysis (NCA) was performed using a commercially available software program (Phoenix WinNonlin V8.3, Certara, Princeton, NJ). For determination of pharmacokinetic parameters in all matrices, concentration data was truncated due to a prolonged terminal phase with extremely low concentrations (see Supplementary Fig. 1). For plasma data, concentrations up to 96 h were utilized. For serum, red blood cells, and whole blood, concentrations up to 9 days were utilized. Plasma concentrations were uniformly weighted for pharmacokinetic analysis. The area under the curve extrapolated to infinity (AUC_inf_) was calculated using the linear up log down method with the extrapolated portion calculated using the formula:

$$= \raisebox{1ex}{${Concentration}_{last}$}\!\left/ \!\raisebox{-1ex}{$lambda\;z$}\right.$$

where lambda z represents the slope of the terminal portion of the curve and includes a minimum of 3 data points.

The percent of the AUC extrapolated (AUC extrap) was calculated using the formula:

$$=100*\left[\raisebox{1ex}{${AUC}_{inf}- {AUC}_{last}$}\!\left/ \!\raisebox{-1ex}{${AUC}_{inf}$}\right.\right]$$

The half-life lambda z was calculated using the formula:

$$= \raisebox{1ex}{$ln(2)$}\!\left/ \!\raisebox{-1ex}{$lambda\;z$}\right.$$

The maximal concentration (C_max_) and time of maximal concentration (T_max_) were determined directly from the concentration curves.

## Results

### In vitro assessment of matrix comparability (Plasma vs Serum)

The % relative difference in metformin concentrations between plasma and serum and serum collected in tubes with and without a separator was < 15% (Tables 2 and 3).

**Table 2:** Differences in metformin concentrations between plasma and serum as determined in vitro

	Plasma %CV	Serum %CV	% relative difference^*^
QC1 (40 ng/mL)	1.9	1.9	0.54
QC2 (5000 ng/mL)	4.3	4.6	2.63

^*^Plasma used as the reference

**Table 3:** Differences in metformin concentrations between serum collected with and without a serum separator as determined in vitro

	Serum %CV	SST CV	% relative difference^*^
QC1 (40 ng/mL)	1.9	1.8	1.93
QC2 (5000 ng/mL)	4.6	3.1	3.23

^*^Serum used as the reference

### Metformin pharmacokinetic study

At approximately 4 h post drug administration, two horses showed mild signs of abdominal discomfort (pawing and rolling); these signs resolved on their own within 2 h. On day 3 (72 h) after beginning the study, one horse was administered oral sulfamethoxazole and trimethoprim for treatment of a condition unrelated to the nature of the present study. Treatment lasted for 14 days. Samples collected from this horse were analyzed for determination of metformin concentrations and were included in the pharmacokinetic analysis.

**Fig. 2:**
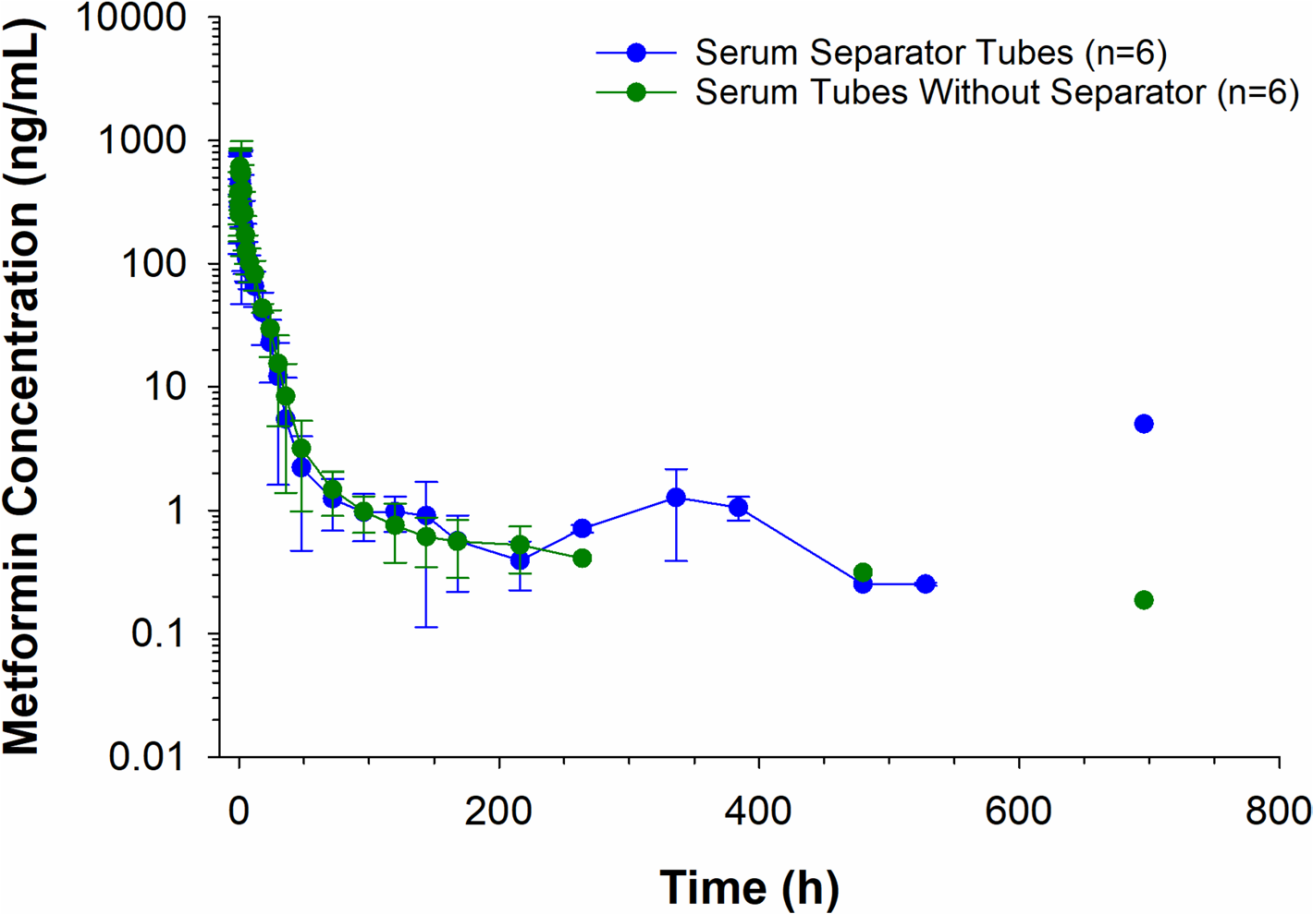
Serum metformin concentrations (mean ± SD) following a single oral administration of 15 g to 6 horses

Serum concentrations (mean ± SD) of metformin are depicted in Fig. 2. Samples were collected into tubes with and without a serum separator for comparison purposes. Metformin concentrations were > 15% different between the 2 tube types at 15 min (16.6%), and 18 (15.8%), 30 (17.0%), 120 (23.9%), 144 (113.9%), and 168 (35.7%) hours (Supplementary Table 1). At all times, where the concentration difference was > 15%, the concentrations in the SST were higher. Metformin serum concentrations were below the LOQ in all horses at 24 and 27 days but were again detectable in one horse at 29 days (Fig. 2).

**Fig. 3:**
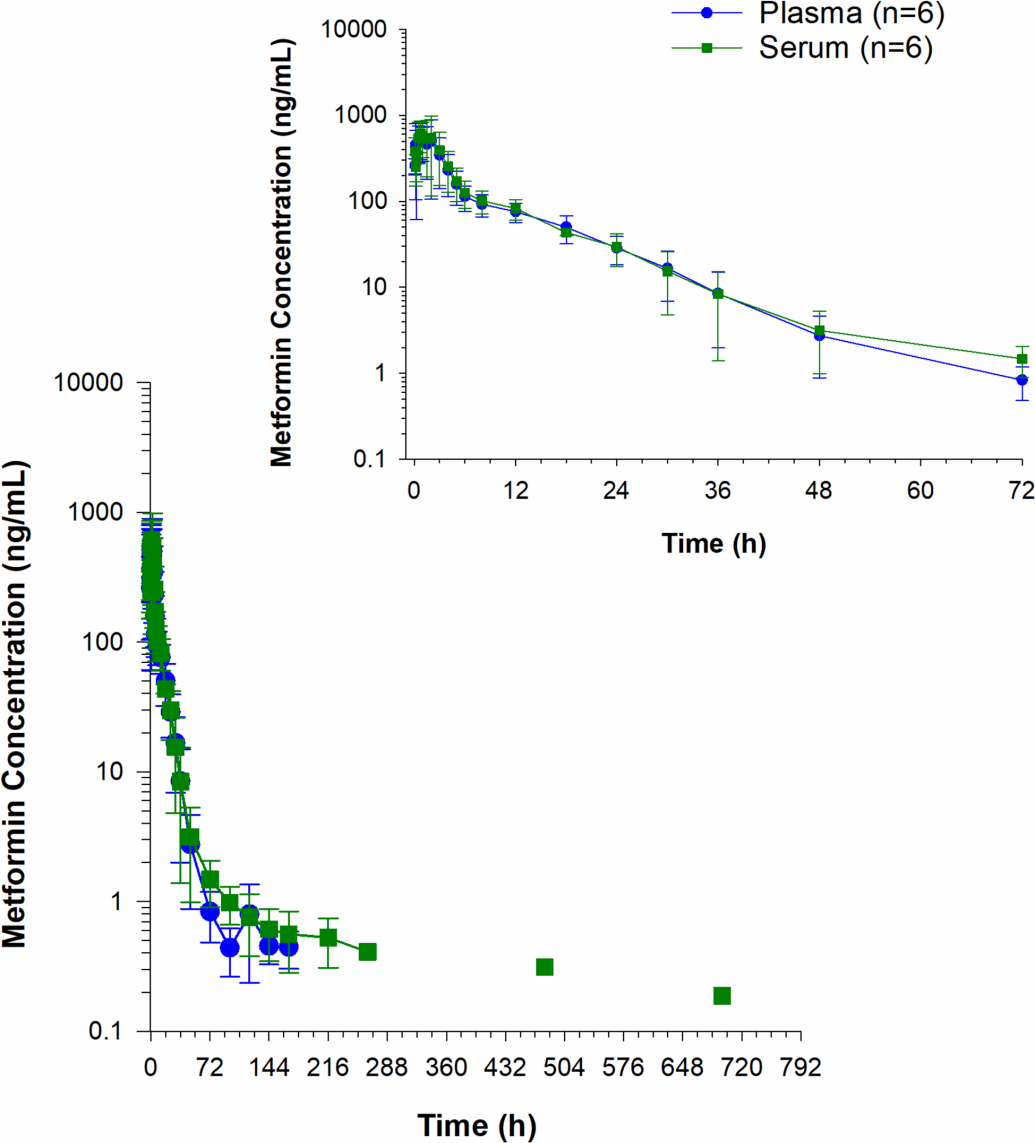
Serum and plasma metformin concentrations (mean ± SD) following a single oral administration of 15 g to 6 horses. Inset represents the first 72 h post drug administration

**Fig. 4:**
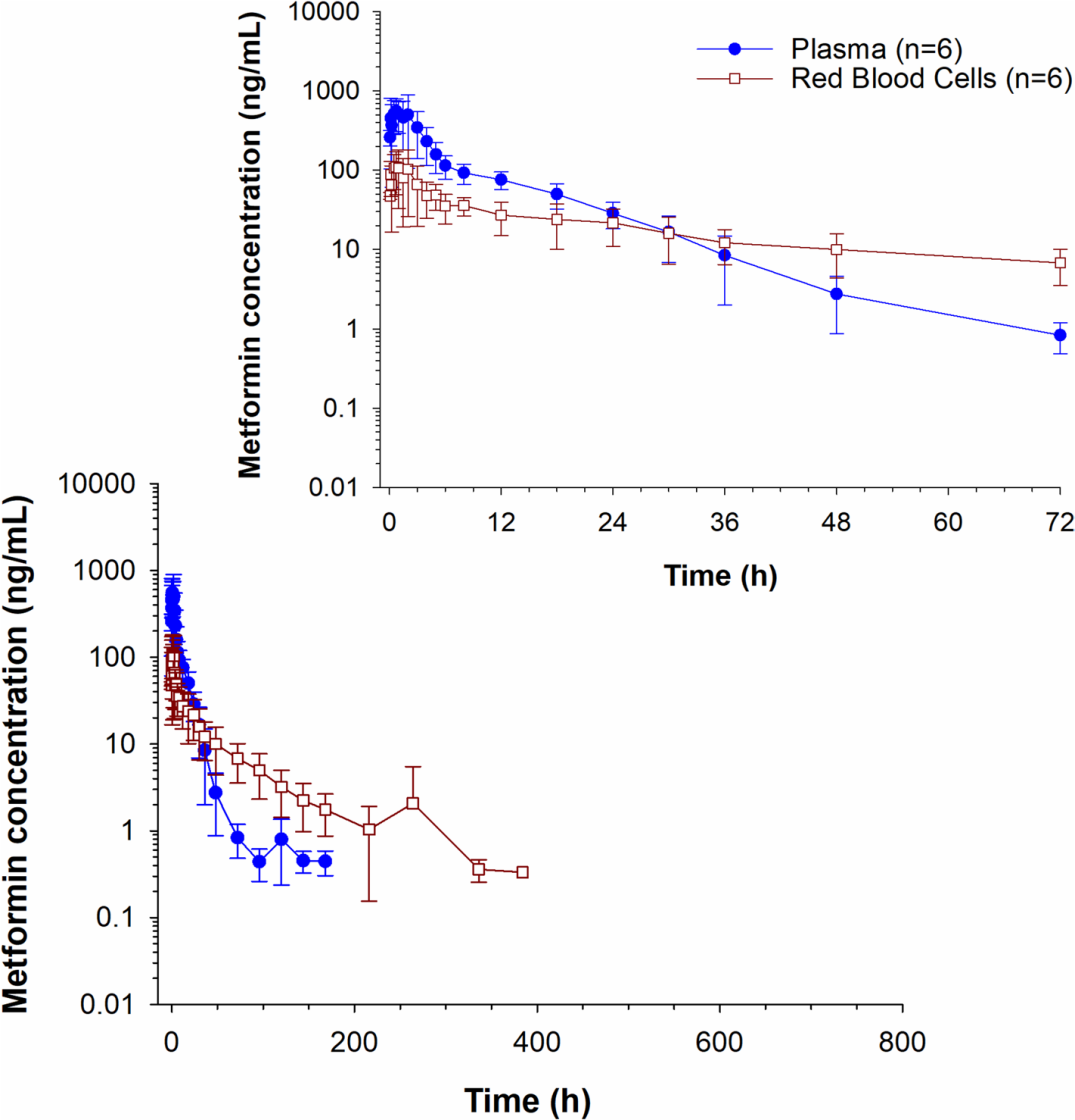
Metformin concentrations (mean ± SD) in plasma and red blood cells (RBC) following a single oral administration of 15 g to 6 horses. Inset represents the first 72 h post drug administration

**Fig. 5:**
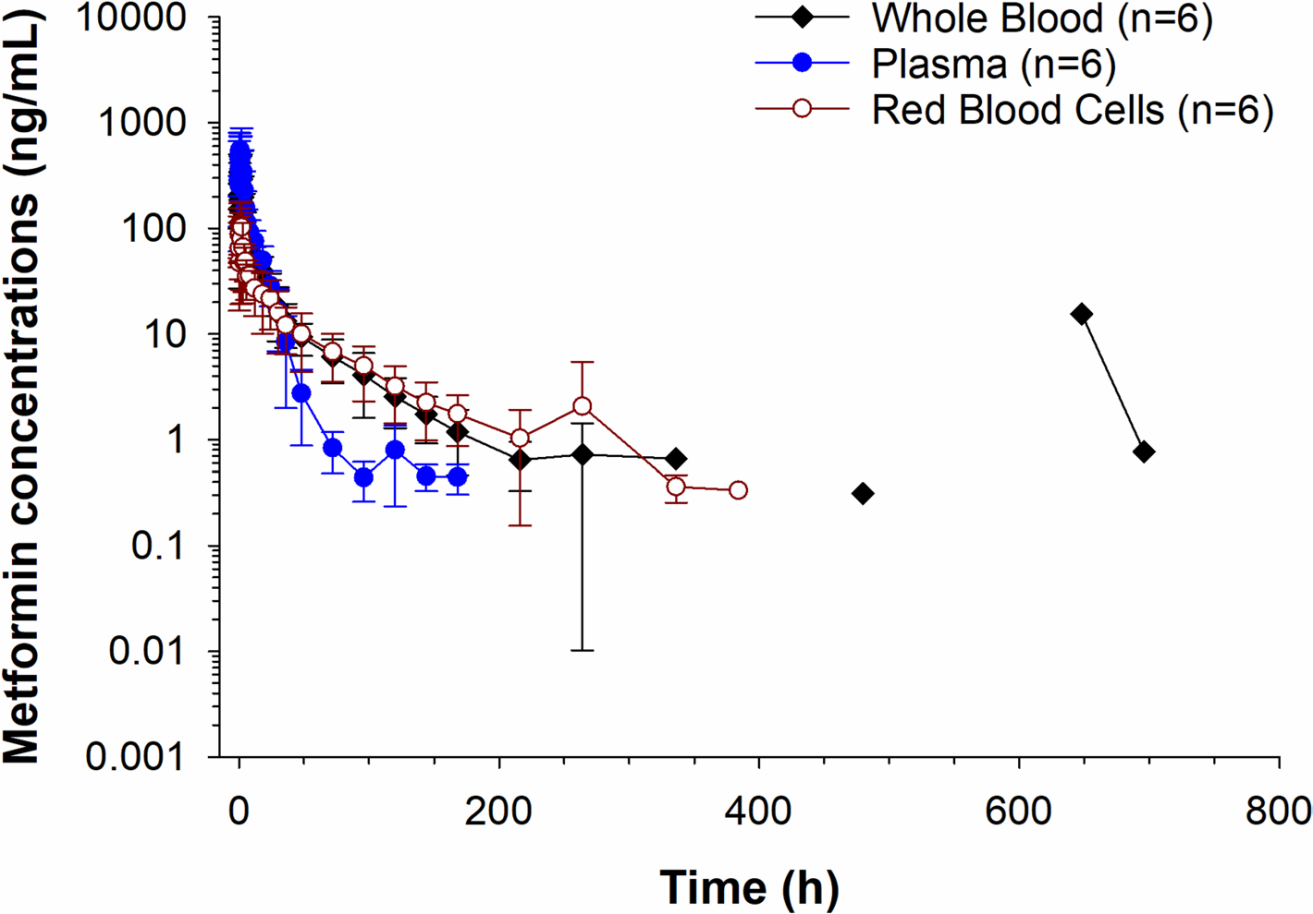
Metformin concentrations (mean ± SD) in whole blood, plasma, and red blood cells following a single oral administration of 15 g to 6 horses

Plasma metformin concentrations over time are depicted in Fig. 3. Metformin was not detected in any of the plasma samples collected between 9 and 14 days. Drug was detected in plasma samples from one horse on day 16 (1.53 ng/mL) and then in a different horse again on day 29 (1.80 ng/ml) and day 31 (0.42 ng/mL). The percent difference between plasma and serum metformin concentrations are reported in Supplementary Table 1. Concentrations were higher in plasma compared to serum (25.6% higher at 10 min; Supplementary Table 1). Serum concentrations were > 15% higher, compared to plasma at 3 (25.1%),4 (31.4%), and from 48–168 h (16.1–150.5%) post drug administration (Supplementary Table 1). Metformin was detected on days 9 and 11 in serum but not plasma (Supplementary Table 1). Metformin concentrations in plasma were higher than RBCs until 24 h post administration. After 24 h, concentrations remained higher in RBCs compared to plasma (Fig. 4). A comparison of metformin concentrations in plasma, RBCs and whole blood at each sample collection time post administration are depicted in Fig. 5. In whole blood samples, metformin concentrations fell below the LOQ in all horses between 14 and 16 days and were detectable again at 20 days (0.32 ng/mL) in one horse. Between 20 and 24 days, concentrations were non-detectable in all horses, returning to detectable concentrations on day 27 (15.5 ng/mL) and day 29 (0.77 ng/mL) in one horse (same horse that had detectable concentrations at 20 days). The B/P ratio for each time point is listed in Table 4. The B/P ratio was less than 1 until the 24-h timepoint. The B/P ratio at the 30-h time point was > 1 with the highest value observed at 96 h (10.6 ± 4.63).

**Table 4:** Metformin concentrations in whole blood and plasma and the blood to plasma (B/P) ratio following a single oral administration of 15 g to six horses. Values are mean ± SD

Time (h)	[Metformin] (ng/mL)		
	Whole Blood	Plasma	B/P ratio^*^
0	ND	ND	–-
0.08	206.6 ± 107.5	258.8 ± 55.7	0.77 ± 0.23
0.16	264.2 ± 237.2	453.3 ± 349.4	0.61 ± 0.33
0.25	152.8 ± 48.6	366.4 ± 305.4	0.53 ± 0.18
0.5	311.2 ± 105.3	516.4 ± 233.4	0.66 ± 0.27
0.75	342.0 ± 156.4	553.7 ± 245.3	0.62 ± 0.05
1	335.4 ± 147.3	512.4 ± 220.2	0.65 ± 0.06
1.5	309.2 ± 193.8	463.1 ± 281.9	0.66 ± 0.03
2	292.7 ± 245.2	499.5 ± 393.4	0.58 ± 0.09
3	198.3 ± 113.5	344.5 ± 203.7	0.58 ± 0.04
4	146.1 ± 66.9	231.2 ± 117.0	0.64 ± 0.05
5	100.8 ± 41.7	158.3 ± 67.3	0.64 ± 0.06
6	68.4 ± 23.6	114.2 ± 37.4	0.60 ± 0.08
8	62.4 ± 14.7	92.8 ± 26.7	0.68 ± 0.06
12	50.8 ± 15.6	75.8 ± 19.0	0.67 ± 0.09
18	39.7 ± 14.2	50.0 ± 17.7	0.80 ± 0.12
24	26.1 ± 11.3	28.8 ± 10.6	0.90 ± 0.08
30	18.1 ± 9.53	16.6 ± 9.76	1.16 ± 0.24
36	13.4 ± 5.97	8.45 ± 6.45	2.08 ± 1.09
48	9.41 ± 3.17	2.75 ± 1.87	4.42 ± 2.19
72	6.14 ± 2.70	0.83 ± 0.35	7.83 ± 3.39
96	4.12 ± 2.51	0.44 ± 0.18	10.6 ± 4.63
120	2.55 ± 1.26	0.80 ± 0.56	7.23 ± 6.98
144	1.73 ± 0.81	0.45 ± 0.13	4.73 ± 2.15
168	1.19 ± 0.73	0.44 ± 0.14	3.99 ± 1.15
Day 9	0.64 ± 0.31	< LOQ	–-
Day 11	0.60 ± 0.71	< LOQ	–-

*LOQ* limit of quantitation, *ND* not detected, not determined

^*^B/P whole blood concentration/plasma concentration

**Fig. 6:**
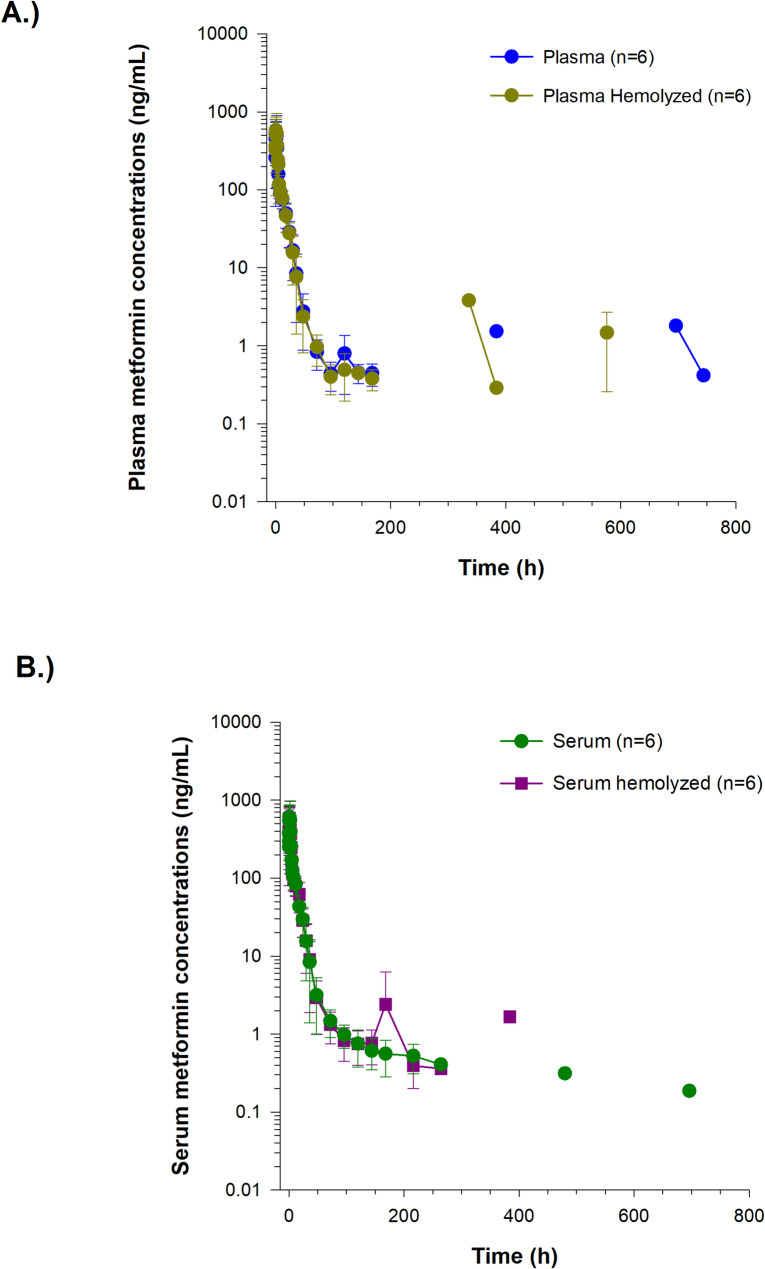
Metformin concentrations (mean ± SD) in hemolyzed and non-hemolyzed (**A**) serum and (**B**) plasma samples following a single oral administration of 15 g 6 horses

Metformin concentrations in hemolyzed serum and plasma samples are compared to non-hemolyzed samples in Fig. 6. Metformin concentrations were > 15% higher in the 72-h hemolyzed plasma sample compared to non-hemolyzed plasma samples. In 2 horses, metformin was detected at 16 and 24 days post administration in hemolyzed plasma samples, whereas drug was not detected in the corresponding non-hemolyzed samples (Fig. 6A). Metformin concentrations were > 15% higher in hemolyzed serum samples compared to non-hemolyzed serum samples at 5 min (480.2 ± 203.6 ng/mL vs 380.7 ± 171.6 ng/mL (mean ± SD) and 10 min (338.5 ± 258.5 ng/mL vs 250.6 ± 99.4 ng/mL) and 18 h (61.8 ± 26.1 ng/mL vs 43.4 ± 3.59 ng/mL) and 144 h (0.77 ± 0.37 ng/mL vs 0.61 ± 0.26 ng/mL) post drug administration. In one horse, metformin was detected at 9 and 11 days post administration in hemolyzed serum samples, whereas drug was not detected in the corresponding non-hemolyzed samples (Fig. 6B).

The mean ± SD, median, and range of select pharmacokinetic parameters following noncompartmental analysis for serum, plasma, RBCs, and whole blood are listed in Table 5. The C_max_ (mean ± SD) of metformin was highest in serum (788.0 ± 289.2 ng/mL) and plasma (800.0 ± 352.2 ng/mL) (Table 5). The C_max_ was 148.7 ± 54 ng/mL and 478.0 ± 209.8 ng/mL for RBCs and whole blood, respectively. The T_max_ was similar between matrices (Table 5). The number of data points used in the calculation of lambda z was 7.3 ± 5.9, 8.0 ± 5.7, 7.2 ± 2.6, and 5.3 ± 1.6 for serum, plasma, RBCs, and whole blood, respectively. The terminal half-life of metformin (mean ± SD) was shortest in plasma at 14.7 ± 7.25 h (Table 5). The terminal half-life was 49.1 ± 7.01, 50.6 ± 15.6, and 75.2 ± 32.2 h for RBCs, whole blood, and serum (Table 5). The terminal half-lives for the horse that received sulfamethoxazole and trimethoprim was within the range reported for other horses (serum: 55.8 h; plasma: 10.0 h; RBCs: 51.3 h).

**Table 5:** Pharmacokinetic parameters for metformin in various blood matrices following a single oral (15 g) administration to 6 horses. Parameters generated using non-compartmental analysis

	C_max_ (ng/mL)	T_max_ (h)	AUC_inf_ (h*ng/mL)	AUC extrap (%)	Lambda z (1/h)	Terminal t_1/2_ (h)
Serum						
Mean ± SD	788.0 ± 289.2	1.0 ± 0.79	4012.3 ± 1344.1	4.72 ± 6.41	0.013 ± 0.010	75.4 ± 32.2
Median	732.7	0.63	3725.9	1.76	0.008	85.8
Range	527.3–1270.5	0.25–2.0	2892.9–6626.5	0.24–16.9	0.006–0.033	20.9–110.8
Plasma						
Mean ± SD	800.0 ± 325.2	0.90 ± 0.70	3592.6 ± 1358.7	0.45 ± 0.40	0.058 ± 0.028	14.7 ± 7.25
Median	695.2	0.63	2999.2	0.30	0.054	13.5
Range	512.6–1262.5	0.16–2.0	2590.3–6130.6	0.15–1.23	0.026–0.103	6.71–27.1
RBCs						
Mean ± SD	148.7 ± 54.1	0.83 ± 0.63	2031.7 ± 917.9	5.57 ± 9.85	0.014 ± 0.002	49.1 ± 7.01
Median	140.0	0.63	1762.5	1.55	0.014	51.2
Range	100.3–247.7	0.25–2.0	1442.6–3788.9	0.90–25.6	0.012–0.019	36.8–55.9
Whole Blood						
Mean ± SD	478.0 ± 209.8	0.76 ± 0.69	3417.2 ± 1456.8	4.59 ± 8.22	0.015 ± 0.003	50.6 ± 15.6
Median	402.2	0.63	2787.4	1.02	0.015	45.5
Range	285.0–764.6	0.16–2.0	2288.2–5841.7	0.60–21.3	0.008–0.017	40.4–81.6

*C*_*max*_ maximum drug concentration, *T*_*max*_ time of maximum drug concentration, *AUC* *inf* area under the curve extrapolated to infinity, *AUC % extrap* percent of area under the curve extrapolated, *Lambda z*, terminal slope of the concentration time curve, *HL lambda z*, terminal half-life

**Fig. 7:**
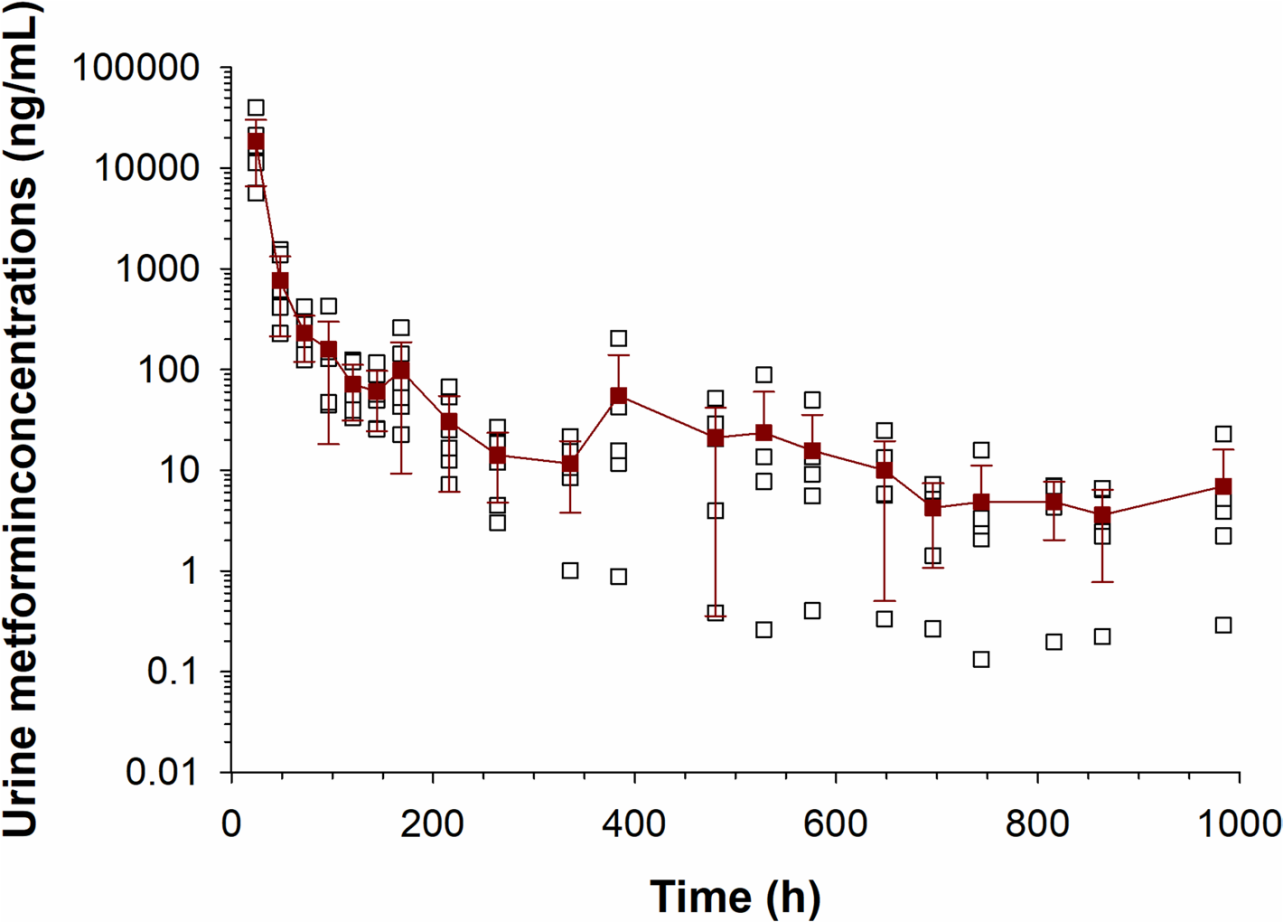
Urine metformin concentrations following a single oral administration of 15 g to 6 horses. Mean concentrations are shown with solid points (± SD), and individual points are shown with open squares

Metformin urine concentrations (mean ± SD) with respect to time are depicted in Fig. 7. Metformin concentrations fluctuated over time for each horse. At day 41 post administration (the last time point collected), urine metformin concentrations in 5/6 horses were above the LOQ.

## Discussion

A previous study conducted by our laboratory demonstrated a prolonged terminal half-life for metformin following administration to horses [6]. Studies in humans have described a “deep” compartment that contributes to this prolonged elimination [2, 7, 11]. This deep compartment includes RBCs [2, 7, 11] and sequestration in these cells creates a depot for the drug, contributing to a prolonged terminal elimination phase [7]. One of the objectives of the current study was to assess whether a similar “deep” compartment exists in horses, perhaps explaining the prolonged terminal half-life reported previously [6].

Serum pharmacokinetic parameters, including C_max_ and T_max_ were consistent with the previous report describing the pharmacokinetics of metformin in horses following oral administration of the same dose [6]. The prolonged terminal serum half-life in the current study (75.8 ± 32.2 h) was also in agreement with the previous report (85.8 ± 15.1 h) [6].

Maximum metformin concentrations in RBCs were lower compared to serum and plasma, similar to what has been reported in humans [7]. In contrast to human studies, whereby maximum concentrations occurred at 4.7 h post drug administration [7], T_max_ in the current study occurred quickly following drug administration (0.83 h). This is not unexpected as the plasma T_max_ in the current study is also much earlier (0.90 h) compared to the previous study in humans (3.0 h) [7]. Distribution of metformin from plasma to RBCs is a result of passive diffusion and therefore the most rapid rate of diffusion into the RBCs would be expected to correlate with the time at which plasma concentrations are highest [7, 12]. In the current study, equilibration of metformin concentrations between plasma and RBCs occurred between 18 and 24 h post administration, with concentrations in RBCs remaining higher than plasma for the remainder of the sample collection period. The longer detection time resulted in a longer terminal half-life in RBCs (49.1 ± 7.01 h) compared to plasma (14.7 ± 7.25 h). This longer detection time in RBCs and subsequently, longer RBC half-life (23.4 ± 1.9 h) compared to plasma (2.7 ± 0.2 h) has also been reported for humans [7]. Notably, the terminal half-life in both plasma and RBCs was longer in the current study compared to the previous report by Robert and colleagues. While this may be related to species differences, it is also important to be cognizant of differences in sampling protocols and the greater sensitivity of the analytical instrumentation utilized in the current study (plasma LOQ:0.25 ng/mL; RBC LOQ: 0.3 ng/mL) compared to the previous study (plasma LOQ: 20 ng/mL and RBC LOQ: 30 ng/mL) [7].

The blood to plasma (B/P) ratio for a drug describes distribution between RBCs and plasma [13]. In an in vitro study, the B/P ratio for metformin was reported to be 1.2 and 1.4 in humans and rats, respectively [14]. In vivo clinical studies, report B/P ratios ranging from 1 to > 10, depending on the time post administration [2, 11]. Tucker and colleagues reported that the metformin B/P ratio was < 1 between 0 and 4 h post drug administration and > 10 at 24 h [11]. A similar trend was observed in the current study, whereby the B/P was < 1 until 18 h post administration, increasing to a B/P of 8 at 72 h post administration and reaching 10.6 at 96 h. This high B/P ratio is also a likely explanation for the long terminal half-life observed in the current study. It is interesting to note that in one horse, the metformin concentration was 15.1 ng/mL at 648 h. Metformin concentration in the two previous samples collected from this horse were non-detectable. There was nothing notably different about this horse or sample and re-extraction and analysis resulted in a similar concentration. Based on this, the most likely explanation for this finding is sequestration in RBCs.

Another notable finding in the current study were differences in metformin concentrations between serum and plasma. This is especially important in equine drug testing, whereby some disciplines or even different horseracing jurisdictions may use serum, and others may utilize plasma for regulatory purposes. Differences between matrices were considered negligible if the % relative difference remained within ± 15%, ensuring that any observed variation is within the limits of acceptable analytical error in accordance with standard bioanalytical method validation acceptance criteria [10]. While the magnitude and significance of the concentration difference varied, depending on the time of sample collection, there were notable differences between plasma and serum. For drugs that sequester in RBCs, such as metformin, concentrations in serum may be artificially elevated due to liberation of the RBC bound fraction into the serum during the clotting process. Since plasma samples are immediately placed on ice and centrifuged, concentrations represent the drug circulating in the aqueous phase and leakage of drug from RBCs into plasma is less of a concern. In the current study, concentrations of metformin were initially higher in plasma compared to serum (> 15% difference). This is not unexpected as concentrations in plasma at these early timepoints represent levels prior to significant distribution into RBCs and tissue. In the case of serum, the time necessary for the clot to form provides a period of time in which the drug in the liquid phase can continue to interact with the RBCs, if distribution equilibrium has yet to be reached, as is likely at these early timepoints, metformin continues to move into the cells, thereby decreasing serum concentrations. Although at most time points post distribution the difference was < 15%, metformin concentrations tended to be higher in serum compared to plasma, most likely due to “cellular leakage.” The difference between the two matrices was more pronounced (> 15%) at the later times (48 h onwards) when more drug was present in the RBCs. The presence of higher concentrations of metformin in RBCs is supported by the much higher B/P ratio at the later time points. Based on these results, it is important to be cognizant of differences in the matrices utilized in experimental studies versus what is used to regulate metformin in equine performance events. If they are different (i.e. plasma versus serum), comparisons may not be appropriate. An additional consideration related to collection of blood samples for drug testing purposes, especially if serum is the preferred matrix, is that the longer the samples sit prior to centrifugation, the greater the likelihood of “cellular leakage” from RBCs into serum due to cellular lysis [15].

Hemolysis, or the rupture of RBCs and subsequent release of cellular contents can occur during sample collection and handling. In the case of drugs like metformin, that are sequestered in RBCs, hemolysis can lead to artificially elevated concentrations of the drug in serum or plasma. In the case of plasma, hemolysis is most likely during sample collection and in the case of serum, this can occur during collection and/or during the clotting time leading to “cellular leakage”, as discussed above. In the current study, overall hemolysis had a minimal impact on blood metformin concentrations. It is important to note that while all attempts were made to induce maximum hemolysis, that the degree of experimentally induced hemolysis may not represent a real-life scenario.

Similar to the report by Jacobs et al., urine metformin concentrations varied greatly between horses [6]. Also as described previously, decreases in urine concentrations would be followed by subsequent increases in individual horses [6]. Jacobs and colleagues speculated that in addition to well-known factors that can affect urine concentrations (i.e. hydration, status, urinary pH, etc.), that these “spikes” may also be due to slow and/or inconsistent release of drug from sequestration sites, such as RBCs [6]. Results of the current study, demonstrating that metformin does sequester in RBCs in horses, supports this theory.

## Conclusion

Results of the current study demonstrate that, as described for humans, metformin sequesters in RBCs, resulting in a prolonged detection time and long terminal half-life. This sequestration led to differences in metformin concentrations between the different blood matrices (i.e. plasma vs serum). These findings are important as the distribution of drugs to “deep” compartments can lead to a prolonged terminal half-life, ultimately affecting efficacy, toxicity, and in the case of regulation of drugs, the detection time.

## Supplementary Information

Supplementary Material 1.

Supplementary Material 2.

## Data Availability

The datasets used and/or analyzed during the current study are available from the corresponding author on reasonable request.

## Abbreviations

B/P ratio: Blood to plasma ratio
C_max_: Maximum concentration
CV: Coefficient of variation
EDTA: Ethylenediaminetetraacetic acid
FDA: Food and Drug Administration
LC–MS/MS: Liquid chromatography mass spectrometry/mass spectrometry
LOD: Limit of detection
LOQ: Limit of quantitation
NCA: Non-compartmental analysis
RBC: Red Blood Cell
SST: Serum separator tube
T_max_: Time of maximum concentration
