# Supplementary material for: Erythrocyte sequestration of metformin in horses: impact on matrix-specific pharmacokinetics and detection windows
Source: BMC Vet Res. 2026 Apr 16;22:314. doi: 10.1186/s12917-026-05449-0 (PMC13214104; doi:10.1186/s12917-026-05449-0)
Supplement: Supplementary file 1 — Supplementary Material 1. [file 12917_2026_5449_MOESM1_ESM.pdf]

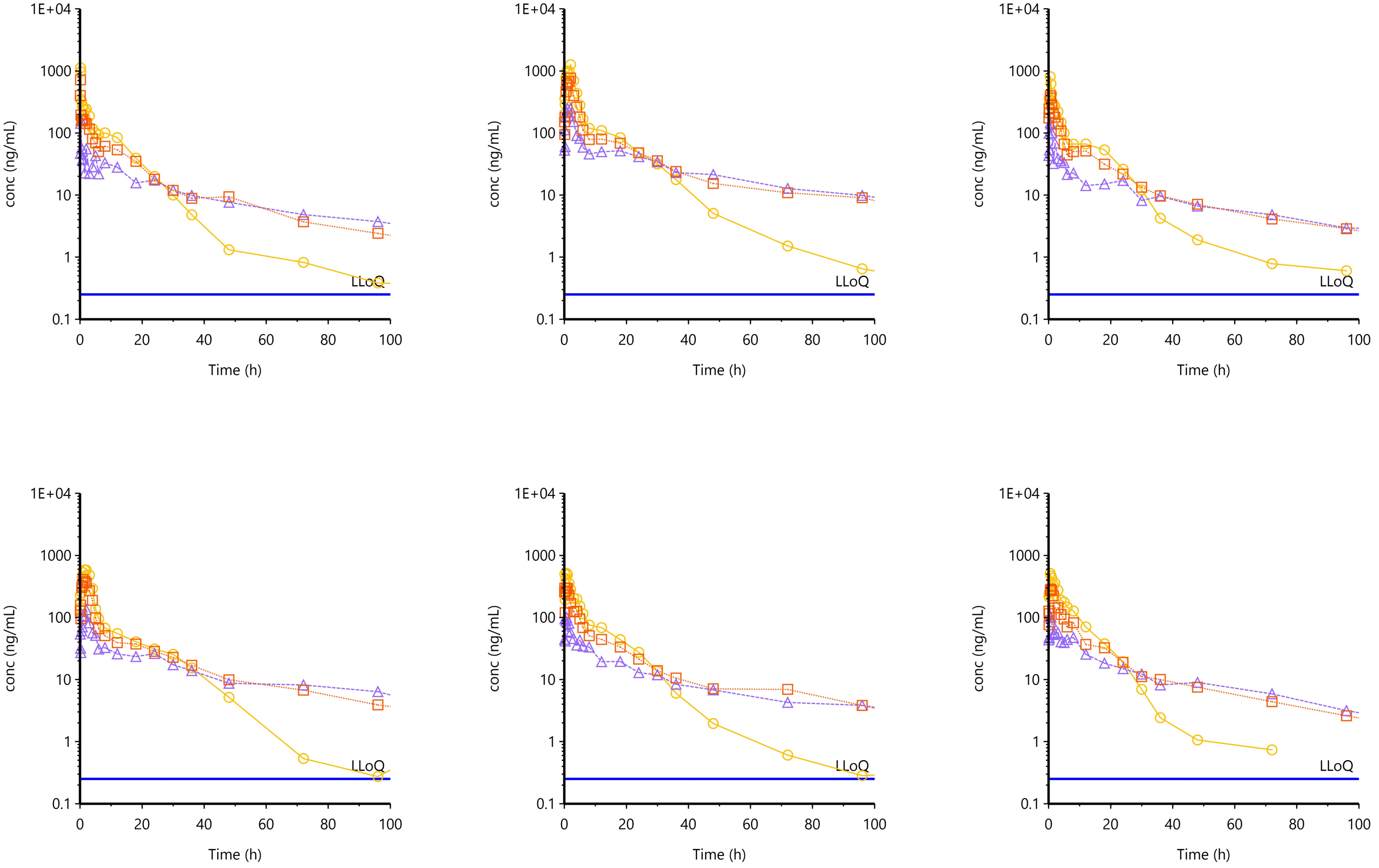

Supplementary Figure 1. Concentration curves including all time points where drug was detected in individual horses. Plasma concentrations are represented by the yellow line, red blood cells by the purple line, and whole blood by the red line.
